# Efficacy of acetaminophen with and without oxycodone for analgesia in non-operative treatment of extremity fractures in adults: protocol for a double-blind randomized clinical trial

**DOI:** 10.1186/s13063-019-3579-x

**Published:** 2019-08-17

**Authors:** Tao Gao, Hongyi Zhu, Changqing Zhang, Yimin Chai, Cheng Guo, Xiaozhong Zhu, Bingbo Bao, Xingwei Li, Junqing Lin, Xianyou Zheng

**Affiliations:** 1 Department of Orthopaedic Surgery, Shanghai Jiaotong University Affiliated Sixth People’s Hospital, NO.600 Yishan Road, Shanghai, China; 2 Department of Pharmacy, Shanghai Jiaotong University Affiliated Sixth People’s Hospital, NO.600 Yishan Road, Shanghai, China

**Keywords:** Opioids, Acetaminophen, Fracture, Non-operative treatment

## Abstract

**Background:**

Opioids and acetaminophen are both widely used to relieve pain after non-operative treatment of limb fractures, but evidence for the superiority of opioids versus acetaminophen is lacking. In this study, we aim to determine whether acetaminophen is non-inferior to the acetaminophen/oxycodone combination for pain relief after non-operative fixation of an extremity limb fracture. We hypothesize that acetaminophen is non-inferior to the acetaminophen/oxycodone combination.

**Methods:**

A double-blind randomized controlled trial will be conducted. Power analysis determined that 1226 participants will be needed (*P* <0.05, power 90%). Patients with acute limb fracture who receive non-operative treatment will be recruited and randomly allocated into two groups: the intervention group will receive oral oxycodone (10 mg) and acetaminophen (650 mg), and the control group will receive acetaminophen (650 mg) only. All participants will be instructed to take one pill of study medication on an as-needed basis and no more frequently than once every 8 h. The primary outcome measure will be scores on the 11-point Numeric Rating Scale (NRS-11) over 14 days. Secondary outcome measures are scores on the Self-Rating Anxiety Scale (SAS), Self-Rating Depression Scale (SDS), EuroQol five-dimension questionnaire (EQ-5D), self-rated satisfaction with the analgesia produced, self-reported nighttime sleep duration, number of intervention or control pills used, and total duration for taking intervention or control medication. Change of pain scores and the number of times that analgesic drugs were taken in the two groups will be statistically evaluated with Student *t* tests according to their fracture site.

**Discussion:**

This study will be a randomized controlled trial that is adequately powered to test the hypothesis that acetaminophen is non-inferior to the combination of acetaminophen and oxycodone in relieving objectively measured pain after non-operative treatment of limb fractures in adults. It will hopefully provide a safe and effective analgesic plan for such patients.

**Trial registration:**

ChiCTR registry: ChiCTR1800017015. Registered on July 8, 2018.

**Electronic supplementary material:**

The online version of this article (10.1186/s13063-019-3579-x) contains supplementary material, which is available to authorized users.

## Background

Several previous studies concluded that opioids were safe and effective and that the benefits outweighed the risks [1]. However, opioid overdose deaths are increasing every year in the US, and it accounted for a great proportion in drug overdose deaths at 60.9% in 2014, 63.1% in 2015, respectively [2–4]. The crisis of opioid abuse is attributed in part to the overprescription of opioid analgesics [5, 6] and highlights the urgent need for evidence on appropriate indication of opioid prescription. A multicenter study in the US showed that 17% of discharged patients in the emergency department (ED) were prescribed opioids and that the incidence of overprescription was high [7].

An epidemiological survey in the US shows that, in the past 20 years, the incidence of fractures has increased by 11%, from 3627 out of 100,000 to 4017 out of 100,000 [8]. It comes mainly from the osteoporosis-related fractures associated with the growth of the elderly population [9] and traffic-related injuries [10]. The periosteum is a highly innervated tissue surrounding the bone tissues, leading to moderate to severe fracture-induced pain [11]. Inappropriate pain management greatly increases the risks of chronic pain [12]. Therefore, appropriate pain relief is important in the management of limb fractures. Opioids and acetaminophen are most widely prescribed to relieve pain in these patients. However, the prescription of these analgesics was supported by little evidence. According to suggestions from the American Academy of Orthopaedic Surgeons, opioids could be prescribed for patients discharged from the ED in consideration of sleeping and life quality. A small-scale trial showed that ibuprofen was as effective as oral morphine but produced fewer side effects for fracture pain in children [13]. A large-scale randomized trial is needed to provide more evidence on this topic, and to eliminate psychological and social bias, there should be blinding with regard to the analgesics used.

Therefore, we plan to conduct a clinical trial to determine whether the analgesic efficacy of acetaminophen is non-inferior to that of oxycodone/acetaminophen for non-operative treatment in adults following acute limb fracture. Our larger aim is to provide a safe and effective analgesic plan for such patients. We hypothesize that acetaminophen is non-inferior to the combination of acetaminophen and oxycodone in relieving objectively measured pain after non-operative treatment of limb fractures in adults.

## Objectives

This study is a single-center, double-blind randomized controlled trial. The purpose of this trial will be to assess the following:

1. whether the analgesic efficacy of acetaminophen is non-inferior to that of oxycodone/acetaminophen for non-operative treatment in adults following acute limb fracture
2. whether the adverse event rate of acetaminophen is lower than that of oxycodone/acetaminophen for non-operative treatment in adults following acute limb fracture.

### Specific primary objective

The specific primary objective is to determine the decline in Numeric Rating Scale (NRS) pain scores from baseline to 1, 3, 7, and 14 days after random assignment to acetaminophen for non-operative treatment in adults following acute limb fracture compared with oxycodone/acetaminophen.

### Specific secondary objectives

The specific secondary objectives are to determine the change in scores on the Self-Rating Anxiety Scale (SAS), Self-Rating Depression Scale (SDS), and EuroQol five-dimension questionnaire (EQ-5D) and the patient’s satisfaction with the medication (0–10) from baseline to 14 days after randomization to acetaminophen for non-operative treatment in adults following acute limb fracture compared with oxycodone/acetaminophen.

### Other secondary objectives

Other secondary objectives are to determine the change in the quality and duration of sleep, number of study medications used, duration that analgesics were taken, and adverse events between the two groups.

## Methods/Design

### Study setting

This study will be performed in the ED of Shanghai Jiaotong University Affiliated Sixth People’s Hospital in China. This hospital is a tertiary and teaching hospital that receives about 350,000 ED visits annually. The study was approved by the ethics committee of Shanghai Jiaotong University Affiliated Sixth People’s Hospital. We used the SPIRIT (Standard Protocol Items: Recommendations for Interventional Trials) reporting guidelines (Additional file 4).

### Study design

**Fig. 1:**
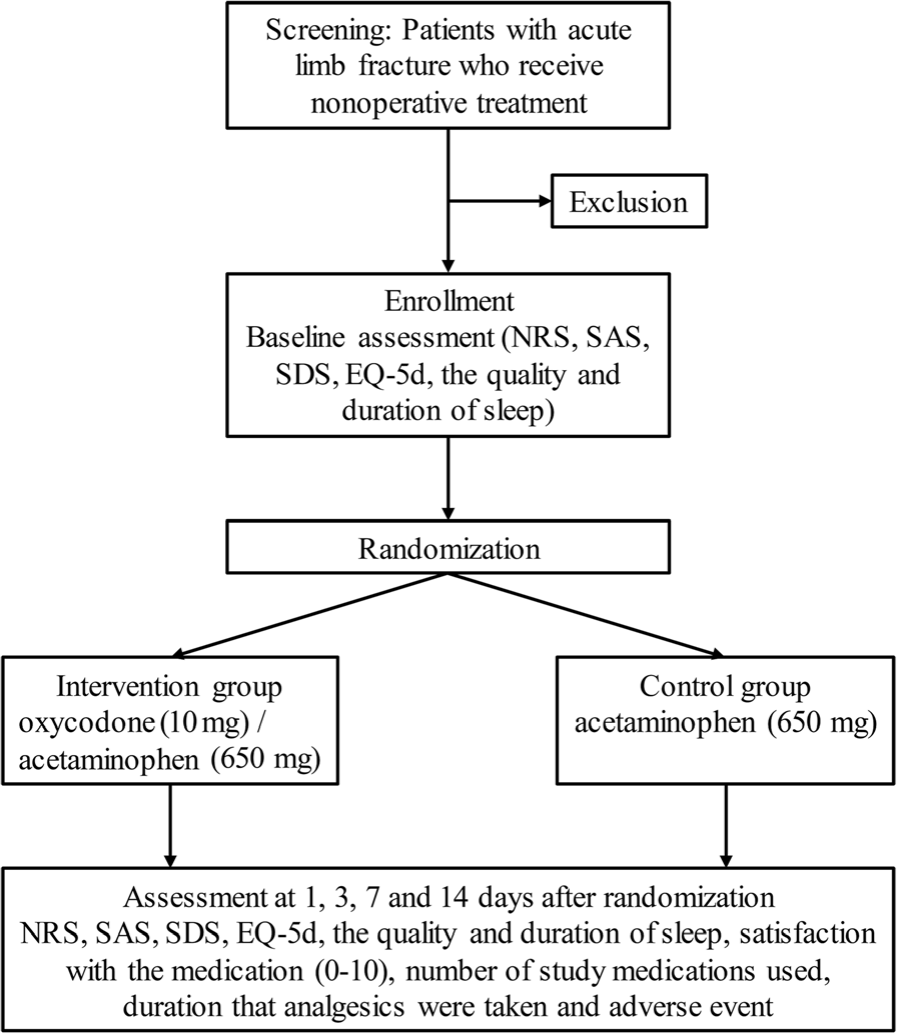
Flowchart of this trial

This study is a single-center, double-blind randomized controlled trial. Figure 1 shows how participants eligible for this trial flow through the study from recruitment to follow-up. Adult patients who have a single acute limb fracture occurring less than one day after injury and who are indicated for non-operative treatment will be recruited. They will be randomly assigned to either the intervention or control group. The intervention group will receive oral pills of oxycodone (10 mg) and acetaminophen (650 mg), and the control group will receive pills of acetaminophen (650 mg) only.

The primary outcome measure will be the 11-point Numeric Rating Scale (NRS-11) [14]. Secondary outcomes include SAS [15], SDS [16], EQ-5D [17], self-rated satisfaction with the analgesia produced by the intervention or control medication (scale of 0–10), self-reported nighttime sleeping duration, total number of analgesic intervention or control pills used during the trial, and total duration for taking intervention or control medication. Any remaining analgesic medications will be collected by the researchers at the end of the trial. Demographic information will be collected from the medical record.

### Eligibility criteria

Adult participants will be recruited prospectively and sequentially as they are admitted to the ED of Shanghai Jiaotong University Affiliated Sixth People’s Hospital. Informed written consent will be obtained from all participants. All methods and procedures in this study are in accordance with the Declaration of Helsinki [18].

### Inclusion criteria

Male and female patients, 18 to 100 years old, will be candidate participants. We will include patients who have an acute limb fracture diagnosed less than one day after injury. Eligible locations of fractures include foot, ankle, tibia, fibula, knee, femur, hip, hand, wrist, forearm, elbow, humerus, shoulder, or clavicle. All candidate participants indicated for non-operative treatment and willing to participate in this study will be enrolled.

### Exclusion criteria

Patients with non-limb fractures or those who have multiple fractures involving more than one site will be ineligible. Also, patients with vascular, nerve, or tendon injuries and open fractures will be excluded. Patients with a pre-injury chronic condition requiring frequent pain management, such as sickle cell disease, fibromyalgia, or any neuropathy, are not eligible, nor are those who have taken methadone at any time in their life. Patients who are pregnant, verified by urine or serum human chorionic gonadotropin testing, will be excluded. Patients who report any adverse reaction or who are allergic to any of the study medications will be dropped from the study. Patients who have contraindications, such as peptic ulcer disease, or who report any prior use of recreational narcotics will be excluded.

Additional exclusion criteria are as follows: medical conditions that might affect metabolism of opioid analgesics or acetaminophen, such as hepatitis, renal insufficiency or failure, hypo- or hyperthyroidism, and Addison’s or Cushing’s disease; taking any medicine that might interact with any of the study medications, such as anticholinergic drugs, oral contraceptives, loop diuretics, probenecid, or liver enzyme inducers; unable to communicate properly and answer questions (e.g., patients with a diagnosis of dementia); physically handicapped people with mobility problems; people with no fixed domicile and short-term visitors who cannot be easily located; and people unwilling or unable to cooperate with data collectors or researchers.

### Randomization and blinding

Stratified blocked randomization will be used to group the participants randomly. The fracture site will be a stratification factor with the following blocks: (1) foot, ankle, tibia, or fibula; (2) knee, femur, or hip; (3) hand; (4) wrist or forearm; (5) elbow or humerus; and (6) shoulder or clavicle. The randomization will be carried out by an independent research assistant, who will not be involved in the enrollment, intervention, or assessment of the participants or data analysis; 1226 unique eight-digit random numbers will be generated for each group (total of six groups) by SAS^®^ version 9.4 software (SAS Institute Inc., Cary, NC, USA). The participants will be randomly assigned to two groups (A and B) in a 1:1 ratio according to the designated interval (only the research assistant will know the interval), and the 1226 random numbers will be randomly assigned to the members of each group. Patients of each group will be further categorized according to fracture site and then the assignments will be documented. These six documents (also referred to as the blinding code) contain the participants’ serial numbers (according to their order of enrollment), random number sequences, and study group assignment; these will be recorded in duplicate and submitted to the clinical research–responsible unit and the research assistant for secure storage. Simplified versions of these documents that do not contain the study group will serve as a reference for allocating study medications to participants.

### Drug preparation

ach study drug package contains one bottle, and each bottle contains 20 tablets of either oxycodone/acetaminophen or acetaminophen tablets (according to the blinding code). The immediate-release oxycodone will be used in this study. The drugs will be contained within identical unmarked, opaque gel capsules. The capsules will be topped up with small quantities of lactose to equalize the weight of the capsules. The shape of the outer packaging and the bottle is exactly the same. A random number sequence will be attached to the outer packet and attached to this will be a sealed opaque envelope (which is called the “emergency letter”), which contains study group allocation and medication type contained within the packet. In the event of an emergency or serious adverse reaction or if the participant needs to know what kind of treatment he or she is receiving, researchers and the participant can obtain the information from the emergency letter. If the seal to the emergency letter is opened and letter is read, the participant will be excluded from the trial.

After data collection is completed, a two-step method is used to reveal group membership. The first step lists only the treatment group to which each participant belongs (such as group A or B) but does not indicate which group is the intervention group or the control group. The second step reveals the treatment received by groups A and B and which group is the control group. The statistical data analyst will also be blinded to participant allocation.

### Intervention

After recruitment, each participant will receive a bottle of tablets according to the random number sequence. The outer package marked with the random number will be tagged with the subject’s serial number, which will be securely stored by researchers for 14 days until the participant’s trial is completed.

All participants will be instructed to take one pill of study medication on an as-needed basis but to take it no more frequently than once every 8 h. Any analgesic other than unblinded oxycodone, which is administered as a rescue analgesic, is prohibited during the trial. We will complete his or her follow-up unceasingly once a participant takes a rescue analgesic, and some extra outcomes, including the number of rescue analgesic used and total duration for taking this medication, will be recorded. Participants will be encouraged to complete follow-ups, and they will have access to the research team anytime to discuss any issues or concerns during the trial. It will be explained to them that they can discontinue the intervention at any time during the trial. Demographic information of participants who withdraw from the study after randomization and their reasons for withdrawal will be collected.

### Assessment

Pain intensity will be assessed by an 11-point numeric rating scale (NRS-11), where “0” is no pain and “10” is the worst possible pain imaginable. Administration of the NRS-11 can be conducted over the phone or in person by a hospital staff data collector. By contrast, Visual Analogue Scale (VAS) scoring can be administered only during in-hospital visits. The NRS-11 is as effective as the VAS in assessing acute pain [19].

Anxiety, depression, and health status and quality of life will be assessed with the following assessment tools. The SAS is a 100-point test (20 items) that assesses the degree of anxiety. A higher score means greater anxiety. The SDS is also a 100-point test (20 items). It assesses the degree of depression. A higher score means that the patient is more depressed. The EuroQol (EQ-5D) is a health description questionnaire that assesses health status and quality of life. It contains two questionnaires comprising five dimensions (mobility, self-care, usual activities, pain/discomfort, and anxiety/depression). Along with the EQ-VAS, EQ-5D can evaluate patients’ health status and quality of life.

### Outcomes

The primary outcome will be the between-group difference in decline in NRS pain scores from baseline to 1, 3, 7, and 14 days after randomization. Secondary outcomes include the between-group difference in change in scores on the SAS, SDS, and EQ-5D from baseline to 14 days after randomization and on a custom in-house developed question on the patient’s satisfaction with the medication (0–10) at 14 days after randomization. For the latter, “0” is very dissatisfied and “10” is very satisfied. Other secondary outcomes are the between-group difference in change in the quality and duration of sleep, number of study medications used, duration that analgesics were taken, and adverse events.

### Data collection

Additional file 1 presents a complete summary of data collected during the trial. Baseline information will be obtained from the participant, including demographic characteristics, sleep quality during the most recent month at the follow-up, and medical history before he or she is recruited. General medical examinations will be also conducted by the researcher. The results of the NRS scores will be reported by participants at 0, 1, 3, 7, and 14 days after randomization. The SAS, SDS, and EQ-5D questionnaires will be completed by participants at 0 and 14 days after randomization during in-hospital visits. A nighttime sleeping diary will be recorded by the participants and then submitted to researchers at 14 days after randomization. Participants will record the number and the times that analgesic drugs were taken. Adverse events will also be recorded throughout this trial. At the end of the trial, we will ask participants which analgesic drugs they believe they took and record it.

### Sample size

The sample size was calculated by using the following parameters. An overall two-sided significance level of 0.05 and power of 90% will be calculated. Referring to previous studies, only if the between-group change difference of NRS scores reached 1.3 units or greater, it seems as clinically significant [14], and the standard deviation of the difference in NRS scores between the two groups was 6.4 points from our prior work. Using these parameters, we calculated a primary sample size of 511 patients per group, giving a total of 1022 patients. Given factors like patients lost to follow-up and participant withdrawal during the trial, it is reasonable to increase the sample size by 20%. Therefore, a total of 1226 participants will be recruited.

### Statistical analysis

All analyses will be carried out by using SPSS version 16.0 (SPSS Inc., Chicago, IL, USA, released 2007, SPSS for Windows, version 16.0, SPSS Inc.). We will perform an intention-to-treat analysis including all randomly assigned participants regardless of whether they obeyed the allocation. Baseline characteristics will be summarized by treatment groups. The results from the trial will be presented as comparative summary statistics (difference in proportions or means) with 95% confidence intervals.

Changes in NRS pain scores (primary outcome); SAS, SDS, and EQ-5D scores; sleep loss; and the number of times analgesic drugs were taken in the two groups will be compared with Student *t* tests, according to their respective fracture site. The incidence of adverse events in the two groups will be presented as proportions and will be compared by using chi-squared/Fisher’s exact test.

### Safety and adverse event reporting

We will record all adverse events observed by researchers or participants that occur within the 14 days of randomization regardless of whether they could be related to the study medication. Once adverse events occur, participants will cease study medications and we will try our best to collect their data. If serious adverse events (SAEs) occur, the investigator will complete the SAE report form immediately and inform the research group within 24 h. If it is not a life-threating event and is thought to be an accident related to the study medication, we will notify the research ethics committee within 15 days. The research ethics committee will be notified within 7 days for a life-threatening event. All of these adverse events will be reported to the ethics review committee at the next meeting. All participants who experienced adverse events will be followed up until the end of the trial.

### Patient and public involvement

In a pilot study, we selected patient representatives to help develop and refine our research project. Because of their complaints about the obvious deterioration of sleep after a fracture, we added “change of sleep quality and duration” as an indicator to evaluate it as a contributor to analgesic efficacy. The idea of treating the fracture sites as a stratification factor also came out of the pilot study.

## Discussions

Opioid-involved overdose deaths are increasing at an alarming rate and this is becoming more common worldwide. Many of these are related to the large amount of opioids being consumed [2–4]. At least for the case of acute pain related to limb fractures, a simple solution would be to reduce opportunities of opioid overconsumption by prescribing an alternative—equally effective—less dangerous analgesic drug. The data to date are confusing and inconsistent about the relative efficacy of opioids and acetaminophen for treating acute pain from limb fractures. These data make it difficult for orthopedic surgeons to implement a safe and effective plan for pain management in non-operative treatment of limb fractures.

Strengths of this study include its large number of study subjects drawn from a large metropolitan hospital specializing in orthopedics, its design as a prospective randomized controlled trial, use of multiple widely used objective measures of experienced pain and anxiety, and the short duration of the trial. Specially, blinding is very important so that all of the outcomes are objective.

The major limitation is that the trial is being conducted at a single health-care center in China. Another limitation is that all outcomes in this study are reported by the patients themselves; thus, there is clearly the potential for reporting bias.

In conclusion, this study is a randomized controlled trial that is adequately powered to test the hypothesis that acetaminophen is non-inferior to the combination of acetaminophen and oxycodone in relieving objectively measured pain after non-operative treatment of limb fractures in adults. At the conclusion of this trial, we will have an answer to the straightforward question: Can fracture-related pain relief be achieved sufficiently with the use of acetaminophen alone?

### Trial status

The trial protocol (version 3.0; May 7, 2018; Additional file 3) was reviewed and approved by the ethics review committee of the Shanghai Jiaotong University Affiliated Sixth People’s Hospital on May 31, 2018. This study started recruiting patients in November 2018. Study recruiting is predicted to end in November 2019.

## Additional files

Additional file 1: Data collection. (DOCX 16 kb)

Additional file 2: Trial registration data. (DOCX 18 kb)

Additional file 3: Trial protocol approved by the institutional review board (IRB). (DOC 73 kb)

Additional file 4: Reporting checklist for protocol of a clinical trial. (DOCX 28 kb)

## Data Availability

The authors will submit the findings of the study to peer-reviewed journals. The raw data obtained in this clinical trial will be disseminated to patients and the public via the Research Manager website (http://www.medresman.org) 6 months after the end of the trial.

## Abbreviations

ED: Emergency department
EQ-5D: EuroQol five-dimension questionnaire
NRS-11: 11-point Numeric Rating Scale
SAE: Serious adverse event
SAS: Self-Rating Anxiety Scale
SDS: Self-Rating Depression Scale
VAS: Visual Analogue Scale
