# Supplementary material for: Efficacy of acetaminophen with and without oxycodone for analgesia in non-operative treatment of extremity fractures in adults: protocol for a double-blind randomized clinical trial
Source: Trials. 2019 Aug 17;20:510. doi: 10.1186/s13063-019-3579-x (PMC6697948; doi:10.1186/s13063-019-3579-x)
Supplement: Supplementary file 1 — Data collection. (DOCX 16 kb) [file 13063_2019_3579_MOESM1_ESM.docx]

Additional file 1: Data collection

| Procedures | Enrolment | Post allocation ( trial) | | | | Close out |
| --- | --- | --- | --- | --- | --- | --- |
|  | V1 | Diary | T1 | T2 | T3 | V2 |
| Eligibility assessment | √ |  |  |  |  |  |
| Informed consent +/- assent | √ |  |  |  |  |  |
| Baseline assessment | √ |  |  |  |  |  |
| Randomization | √ |  |  |  |  |  |
| Allocation of study medications | √ |  |  |  |  |  |
| Adverse events assessment |  |  | √ | √ | √ | √ |
| NRS score | √ |  | √ | √ | √ | √ |
| The Self-Rating Anxiety Scale (SAS) | √ |  |  |  |  | √ |
| The Self-Rating Depression Scale (SDS) | √ |  |  |  |  | √ |
| The EuroQol (EQ-5d) | √ |  |  |  |  | √ |
| Satisfaction with the medication (0-10) |  |  |  |  |  | √ |
| The quality and duration of sleep | √ | √ |  |  |  |  |
| Number of study medications used |  |  |  |  |  | √ |
| Duration that analgesics were taken |  |  |  |  |  | √ |

V1 = Visit 1; V2 = Visit 2 (14 days after randomization); T1 = telephone follow-up 1 (1 day after randomization); T2 = telephone follow-up 2 (3 days after randomization); T3 = telephone follow-up 3 (7 days after randomization).

.
