# Supplementary material for: Efficacy of acetaminophen with and without oxycodone for analgesia in non-operative treatment of extremity fractures in adults: protocol for a double-blind randomized clinical trial
Source: Trials. 2019 Aug 17;20:510. doi: 10.1186/s13063-019-3579-x (PMC6697948; doi:10.1186/s13063-019-3579-x)
Supplement: Supplementary file 2 — Trial registration data. (DOCX 18 kb) [file 13063_2019_3579_MOESM2_ESM.docx]

Additional file 2: Trial registration data

| Data category | Information |
| --- | --- |
| Primary registry and trial identifying number | Chinese Clinical Trial Registry ChiCTR1800017015 |
| Date of registration in primary registry | 8 July, 2018 |
| Source(s) of monetary or material support | Shanghai Jiaotong University Affiliated Sixth People’s Hospital |
| Primary sponsor | Shanghai Jiaotong University Affiliated Sixth People’s Hospital |
| Contact for public queries | Xianyou Zheng [zhengxianyou@126.com] |
| Contact for scientific queries | Xianyou Zheng Shanghai Jiaotong University Affiliated Sixth People’s Hospital, Shanghai, China |
| Public title | Pain management after nonoperative treatment of limb fracture - a double-blind randomized controlled trial |
| Scientific title | *Pain management after nonoperative treatment of limb fracture - a double-blind randomized controlled trial* |
| Countries of recruitment | China |
| Health condition(s) or problem(s) studied | pain management, limb fracture |
| Intervention(s) | intervention group: oral oxycodone (5 mg)/acetaminophen (325 mg), take one pill on an as-needed basis, but no more frequently than once every 4 hours |
|  | control group: oral acetaminophen (650 mg), take one pill on an as-needed basis, but no more frequently than once every 4 hours |
| Key inclusion and exclusion criteria | Ages eligible for study: ≥18 years  Sexes eligible for study: both Accepts healthy volunteers: no |
|  | Inclusion criteria: Aged from 18 to 100 years old, male or female is not limited; diagnosis of acute limb fracture less than one day after injury; location of fractures including foot, ankle, tibia, fibula, knee, femur, hip, hand, wrist, forearm, elbow, humerus, shoulder and clavicle; indicated for nonoperative treatment; and willing to participated in this study. |
|  | Exclusion criteria: With other non-limb fractures; multiple fractures involved more than one site; with vascular, nerve or tendon injuries; open fractures; chronic condition requiring frequent pain management such as sickle cell disease, fibromyalgia, or any neuropathy; have taken methadone ever; report of an adverse reaction to any of the study medications; allergic to any of the study medications or contraindications such as peptic ulcer disease; report of any prior use of recreational narcotics; medical condition that might affect metabolism of opioid analgesics, acetaminophen, such as hepatitis, renal insufficiency or failure, hypo- or hyperthyroidism, Addisons or Cushings disease; taking any medicine that might interact with any of the study medications, such as anticholinergic drugs, oral contraceptives, loop diuretic, probenecid or liver enzyme inducer; history of mental disorders, unable to communicate properly and answer questions such as dementia etc; physically handicapped people with mobility problems; no fixed address and easily lost to visitors; pregnancy by either urine or serum HCG testing; unwilling or unable to cooperate with data collectors. |
| Study type | Interventional |
|  | Allocation: randomized intervention model. Parallel assignment masking: double blind (subject, caregiver, investigator, outcomes assessor) |
|  | Primary purpose: noninferior trial |
|  | other |
| Date of first enrolment | Not yet |
| Target sample size | 1000 |
| Recruitment status | Recruiting |
| Primary outcome(s) | NRS score (time frame: 14 days; not designated as safety issue) |
| Key secondary outcomes | SAS, SDS, and EQ-5d scores, the patient’s satisfaction with the medication, change in the quality and duration of sleep, number of study medications used and duration that analgesics were taken (time frame: 14 days; not designated as safety issue) |
