# Supplementary material for: Efficacy of acetaminophen with and without oxycodone for analgesia in non-operative treatment of extremity fractures in adults: protocol for a double-blind randomized clinical trial
Source: Trials. 2019 Aug 17;20:510. doi: 10.1186/s13063-019-3579-x (PMC6697948; doi:10.1186/s13063-019-3579-x)
Supplement: Supplementary file 3 — Trial protocol approved by the institutional review board (IRB). (DOC 73 kb) [file 13063_2019_3579_MOESM3_ESM.doc]

**研究方案**

**项目名称（中文）：**四肢骨折保守治疗后镇痛药物效果研究——一项随机对照双盲试验

**项目名称（英文）：**Pain management after conservative treatment of limb fracture - a double-blind randomized controlled trial

研究单位：上海市第六人民医院

研究负责人：郑宪友

申办者：上海市第六人民医院

1. **研究背景**

随着我国交通、建筑等行业的发展及人口老龄化加剧,导致交通伤、建筑伤和老年骨质疏松性骨折患者快速增加。美国一项流行病学调查显示在过去20年里，骨折发生率增加了11%，从3,627/100,000人口增加到4,017/100,000人口。四肢骨折创伤后由于各种因素（骨折端摩擦、肿胀、心理因素等）导致不同程度的疼痛，早期疼痛不仅明显影响患者的一般状况，还会引起严重的并发症，使病情恶化。因此，对选择保守治疗四肢骨折患者早期进行有效的镇痛治疗显得很有意义。各种镇痛药都有其各自的优缺点，例如阿片类药物镇痛效果好，但是副作用较大；而对乙酰氨基酚镇痛效果略差，但副作用较小。目前对于四肢骨折保守治疗镇痛药物的选择尚无相关的临床试验提供证据作为参考，因此我们进行此项临床试验，为此类患者提供安全有效的镇痛方案。

上海市第六人民医院骨科是国家重点学科，也是卫生部临床重点专科，上海市创伤骨科临床医学中心，并入选上海市“重中之重” 。且经上海市卫计委批准成立上海市急性创伤急救中心，依托强大的骨科临床技术，可以募集并随访到来自不同地区的急性四肢骨折患者，为试验的进行提供了重要的支撑。在数据管理方面，由专门实验人员负责患者信息，对所有数据进行加密处理，切实保护患者的隐私。

**二、研究目的**

进行一项随机对照双盲试验，确定骨折保守治疗患者单独应用对乙酰氨基酚是否不差于应用对乙酰氨基酚/羟考酮复合制剂的镇痛效果。本研究的更大目标是为该类患者提供安全有效的镇痛计划。

**三、研究概况**

3.1整体的研究设计和计划

本研究为一项前瞻性、双盲、随机、对照研究。

3.2 研究人群

主要是由创伤导致的急性四肢骨折且行保守治疗的成年（18-100周岁）患者，并且来自就诊于本单位的患者。

3.2.1入选标准

1. 年龄18-100周岁，性别不限；
2. 少于一天的急性四肢骨折；
3. 骨折部位包括足、踝、胫骨、腓骨、膝、股骨、髋、手、腕、前臂、肘、肱骨、肩与锁骨。
4. 有保守治疗的指征；
5. 有意愿参加本试验。

3.2.2排除标准

1. 合并其他非四肢骨折者；
2. 多部位多发骨折者；合并血管、神经及肌腱损伤者；
3. 开放性骨折者；
4. 需要频繁疼痛管理的慢性病者，如镰状细胞病，纤维肌痛或任何神经性疼痛等；
5. 曾使用过美沙酮者；
6. 对研究药物产生过不良反应者；
7. 对研究药物过敏或禁忌症者，如消化性溃疡；曾使用过娱乐性麻醉品者；
8. 可能影响阿片类药物、对乙酰氨基酚代谢的医疗状态者，如肝炎，肾功能不全或衰竭，甲状腺功能低下或亢进症，艾迪生氏病或库欣病；
9. 正服用任何可能与研究药物相互作用者，例如抗胆碱能药、口服避孕药、髓袢利尿剂、丙磺舒或肝酶诱导剂；
10. 精神疾病史、无法正常交流及回答问题者，例如痴呆等；
11. 肢体残疾行动不便者；
12. 无固定地址易失访者；
13. 血或尿HCG检测显示怀孕者；
14. 不愿意或无法配合资料收集者；

3.2.3剔除标准

1. 试验进程中服用除用作紧急镇痛药的非盲羟考酮以外的其他镇痛药者；
2. 试验进程中服用影响阿片类药物、对乙酰氨基酚代谢的药物者；

3.2.4中途退出标准

1）受试者要求撤回知情同意；

2）研究者从医学角度考虑受试者有必要中止研究。

3.3病例数及分组方法

选择临床上由创伤导致的急性四肢骨折且行保守治疗的成年（18-100周岁）患者1226例，随机分为A组（干预组），613例。予以氨酚羟考酮片（325mg对乙酰氨基酚+5mg羟考酮），患者自行按需服用，常规予以疼痛管理宣教和用量指导教育；B组（对照组），613例，予以对乙酰氨基酚片（650mg对乙酰氨基酚），患者自行按需服用，常规予以疼痛管理宣教和用量指导教育。

3.3试验药物

试验药（泰勒宁（氨酚羟考酮片））：片剂、剂量为325mg对乙酰氨基酚+5mg盐酸羟考酮、口服给药、常规剂量为每4小时服用1片，可根据疼痛程度和给药后反应来调整剂量。

对照药（泰诺林（对乙酰氨基酚））：片剂、剂量为650mg对乙酰氨基酚、口服给药、常规剂量为每4小时服用1片，24小时内不得超过4次。

用随机化方法产生1226个处理编码并随机分配好试验组别后，准备药品（由朱弘一负责随机生成1226个处理编码、装配药物并完成盲底的编制及保存）。每份试验药品包装盒中包含1瓶，每瓶里面放置20片由外观完全一致胶囊装载（由我院中心药房协助完成工作）的泰勒宁或泰诺林片剂（依编码所属试验组别），外包装及瓶子形状完全相同，在外包装上写上对应的处理编码，存档。记录有受试者序号、药物编码、试验组别的文件（亦称为盲底）一式两份，交临床研究负责单位和研究申请人分别保存。在每一份完成编码的试验药物包装盒中设置一份应急信件，内容包括该编号的受试者所分入的组别及用药情况，非必要时不得拆阅。在发生紧急情况或病人需要抢救必须知道该病人接受的是何种处理时，由研究人员按试验方案规定的程序拆阅。一旦被拆阅，该编号病例将中止试验。

数据采集完成后，采用两次揭盲法：第一次揭盲只列出每个病例所属的处理组别( 如A 组或B 组) 而并不标明哪一个为试验组或对照组； 第二次揭盲明确各组所接受的治疗: 例如宣布A ，B中谁是试验组、谁是对照组。

3.4研究步骤及相关检查

筛选期（四肢骨折后行保守治疗服用镇痛药物前），将收集受试者一般人口学资料、生活习惯和既往疾病史并进行一般体格检查；入选治疗期，严格按照入排标准确定受试者是否纳入本研究，获受试者知情同意并签署知情同意书后，对受试者进行NRS评分、填写焦虑抑郁量表、EQ-5d生活质量量表等。通过随机数法对受试者进行随机分组，严格按照分组安排发放试验药物，并遵医嘱服用；随访期（保守治疗后1天），对受试者进行NRS评分、并记录受试者睡眠时间的变化；随访期（保守治疗后3天），对受试者进行NRS评分、并记录受试者睡眠时间的变化；随访期（保守治疗后7天），对受试者进行NRS评分、并记录受试者睡眠时间的变化。随访期（保守治疗后14天），对受试者进行NRS评分、填写焦虑抑郁量表、EQ-5d生活质量量表以及对镇痛效果的满意度评分（0-10）、记录服用镇痛药物的数量和服用镇痛药物时间，睡眠时间和质量的变化以及不良事件。其中第1、2、3次随访采取电话随访形式、末次随访采用现场随访形式。

受试者药物发放由朱晓中、鲍丙波、李星玮、高涛等人进行并完成后续受试者随访工作，于试验开始前进行规范化培训及考核，考核合格后方可进行受试者药物发放以及后续随访、数据收集。

3.5 终点指标

主要终点指标：疼痛治疗前后NRS评分的差值；主要时间节点为疼痛治疗后14天。

次要终点指标：焦虑抑郁量表、EQ-5d生活质量量表、对镇痛的满意度（0-10分）、睡眠时间及质量的变化、服用镇痛药物的数量、服用镇痛药物时间和不良事件。

3.6中止临床研究的标准

1） 发生严重不良事件

3.7 合并用药和治疗

在本研究中，您禁止使用以下药物：中枢神经系统抑制剂、抗胆碱能药、口服避孕药、β受体阻滞剂、髓袢利尿剂、拉莫三嗪、丙磺舒、齐多夫定等。

3.8 研究结束后的随访和医疗措施

研究结束后，若仍有镇痛需要，您可来我院门诊寻求镇痛管理措施。

**四、不良事件观察**

4.1 不良事件的定义

4.1.1定义

不良事件：病人或临床试验受试者接受一种药品后出现的不良医学事件，但并不一定与治疗有因果关系。

严重不良事件：临床试验过程中发生需住院治疗、延长住院时间、伤残、影响工作能力、危及生命或死亡、导致先天畸形等事件。

4.1.2程度

轻度：受试者可忍受，不影响治疗，不需要特别处理，对受试者康复无影响。

中度：受试者难以忍受、需要特殊处理，对受试者康复有直接影响。

重度：危及受试者生命，致死或致残，需立即做紧急处理。

4.2不良事件的记录及报告途径

受试者治疗过程中可能发生临床不良事件，一旦发生不良事件（包括重要不良事件），应在病例报告表上详细记录不良事件的发生时间、临床表现、处理经过和持续时间、转归以及与药物的关系；出现实验室检查异常者，须随访患者至检查结果恢复正常，或至用药前水平，或确定与试验药物无关。发生严重不良事件应填写严重不良事件表，并在24小时内报告申办者、伦理委员会、CFDA安监司和卫生行政部门。

4.3 风险的防范和处理

风险的防范：在受试者签署知情同意书之前充分详细地向受试者解释该试验所用药物可能存在的不良反应及其表现形式，向其宣教该药物的用法用量等；用药期间进行严格随访是否出现不良反应，一旦出现不良反应，无论是否与所用药物有关，均对其进行积极处理。且处理与本研究相关的损害的过程中所发生的费用均由研究者承担。

**五、统计分析**

5.1样本含量估计

样本量计算采用计量资料优效性临床试验的样本计算公式。

即n=2[(U_α_+U_β_)S/δ]^2^

假设本试验预计的病例退出率为20%，α取0.05、β取0.1查表可得到U_α_ (0.05)=1.65，U_β_（0.1）=1.28；治疗前后NRS评分改变量在两组间的差值达1.3分或以上方可视镇痛效果有临床意义；查资料可得两组NRS评分差值共同标准差为6.4分。

U_α_ (0.05)=1.96，U_β_（0.1）=1.282， S=6.4， δ=1.3

代入公式：N_A_=N_B_=2×[(1.96+1.282) ×6.4/1.3]^2^=511

临床研究过程中由于失访，资料不合格等会造成样本量的消耗，所以，在估算的样本量基础上，适当增加20% 样本量，以保证最后纳入分析时能保证足够的样本量。

因此通过计算公式估计本临床试验各分组样本量约为613例，本临床试验所需样本量总数为1226例。

5.2研究数据的统计与分析

试验结束后将研究数据交由第三方统计分析师进行分析。采用SPSS16.0统计软件进行处理，计量资料以均数表示，组间比较采用t检验，P＜0.05为差异有统计学意义。

**六、研究相关伦理学**

6.1伦理委员会审核

本方案和书面知情同意书及与受试者直接相关的资料必须提交伦理委员会，获得伦理委员会书面批准后方可正式开展研究。研究者必须至少每年（如果适用）向伦理委员会提交研究年度报告。在研究中止和/或完成时，研究者必须书面通知伦理委员会；研究者必须及时向伦理委员会报告所有研究工作中发生的变化（如方案和/或知情同意数的修订），并且在未获得伦理委员会批准之前不得执行这些变动，除非是为了消除对受试者明显且直接的风险而做出的变更。在发生这类情况时，将通知伦理委员会。

6.2知情同意

6.2.1获得知情同意的程序

研究者必须向受试者或其法定代理人提供易于理解的并且经伦理委员会批准的知情同意书，并给与受试者或其法定代理人充分的时间考虑本项研究，在从受试者获得签署的书面知情同意书之前，受试者不得入组。 在受试者参与期间，将向受试者提供所有更新版本的知情同意书以及书面信息。知情同意书应作为临床试验的重要文档保留备查。

1. **数据保密和安全监察计划**

通过本项目研究的结果可能会在医学杂志上发表，但是我们会按照法律的要求为患者的信息保密，除非应相关法律要求，患者的个人信息不会被泄露。必要时，政府管理部门和医院伦理委员会及其有关人员可以按规定查阅患者的资料。

1. **临床研究的质量控制与质量保证**

试验开始前，制定临床试验标准操作规程（SOP），对所有的参试人员进行相关培训，并在试验开始的阶段认真检查SOP的执行，在执行中对SOP的适用性和有效性进行系统的检查，对确认不适用的SOP进行修改和补充。

临床试验过程的每项工作都根据有关法规及管理规定、工作职责、该工作的技术规范和该试验方案的要求制定该项工作的标准操作规程。

明确规定试验对象和变量，随机化选择并分配试验对象。采用双盲试验，保证试验组和对照组的均衡性；召集试验人员进行统一技术培训和技术考核。

**Translated version**

**Trial protocol**

Title: Pain management after conservative treatment of limb fracture - a double-blind randomized controlled trial

Institute: Shanghai Sixth People's Hospital

Study leader: Xianyou Zheng

Sponsor: Shanghai Sixth People's Hospital

1. **Background**

With the development of China's transportation and construction industries and the aging of the population, the number of traffic injuries, construction injuries, and elderly osteoporotic fractures has increased rapidly. An epidemiological survey in the United States shows that in the past 20 years, the incidence of fractures has increased by 11%, from 3,627/100,000 to 4,017/100,000. Various factors (fracture friction, swelling, psychological factors, etc.) lead to different degrees of pain after limb fracture. Early pain not only significantly affect the general condition of patients, but also cause serious complications, so that the disease worse. Therefore, it is of great significance to choose early and effective analgesic therapy for nonoperative treatment of patients with limb fractures. All kinds of analgesics have their own advantages and disadvantages. For example, opioids have good analgesic effects, but they have more side effects. Acetaminophens have slightly less analgesic effects but have fewer side effects. At present, there is no relevant clinical trial to provide evidence for the choice of nonoperative treatment of analgesic drugs for limb fractures. Therefore, we conducted this clinical trial to provide a safe and effective analgesic plan for such patients.

The Orthopedics Department of the Shanghai Sixth People’s Hospital is a national key discipline and is also a clinical specialization department of the Ministry of Health, Shanghai Trauma Orthopedic Clinic Medical Center, and was selected as “the top priority” in Shanghai. With the approval of the Shanghai Municipal Health and Family Planning Commission, the Shanghai Emergency Trauma Emergency Center has been established. Relying on powerful orthopedic clinical techniques, we can recruit and follow patients with acute limb fractures from different regions, providing important support for the trial. In terms of data management, specialized laboratory personnel are responsible for patient information and encrypt all data to ensure the privacy of patients.

1. **Objectives**

A randomized controlled double-blind trial was conducted to determine whether acetaminophen alone is comparable to acetaminophen/oxycodone in pain relief after nonoperative treatment of limb fractures. The larger aim is to provide a safe and effective analgesic plan for such patients.

1. **Research Overview**
   1. Overall research design and planning

This study will be a prospective, double-blind, randomized, controlled study.

- 1. Research population

The participants are adult patients (18-100 years old) who received nonoperative treatment after acute traumatic limb fractures, and they visited our hospital.

- - 1. Participant inclusion criteria:

1. Age from 18 to 100 years old, male or female is not limited;
2. Diagnosis of acute limb fracture less than one day after injury;
3. Location of fractures including foot, ankle, tibia, fibula, knee, femur, hip, hand, wrist, forearm, elbow, humerus, shoulder and clavicle;
4. Indicated for nonoperative treatment;
5. And willing to participated in this study.
   - 1. Exclusion criteria
6. With other non-limb fractures;
7. Multiple fractures involved more than one site;
8. With vascular, nerve or tendon injuries;
9. Open fractures;
10. Chronic condition requiring frequent pain management such as sickle cell disease, fibromyalgia, or any neuropathy;
11. Have taken methadone ever;
12. Report of an adverse reaction to any of the study medications;
13. Allergic to any of the study medications or contraindications such as peptic ulcer disease; report of any prior use of recreational narcotics;
14. Medical condition that might affect metabolism of opioid analgesics, acetaminophen, such as hepatitis, renal insufficiency or failure, hypo- or hyperthyroidism, Addisons or Cushings disease;
15. Taking any medicine that might interact with any of the study medications, such as anticholinergic drugs, oral contraceptives, loop diuretic, probenecid or liver enzyme inducer;
16. History of mental disorders, unable to communicate properly and answer questions such as dementia etc;
17. Physically handicapped people with mobility problems; no fixed address and easily lost to visitors;
18. Pregnancy by either urine or serum HCG testing;
19. Unwilling or unable to cooperate with data collectors.
    - 1. Elimination criteria
         1. taking other analgesics other than unblinded oxycodone which is administered as a rescue analgesic during the trial;
         2. taking drugs that affect the metabolism of opioids and acetaminophen during the test;
      2. Standard of withdrawal
20. The participant requested the withdrawal of informed consent;
21. The investigator considers it necessary for the participant to discontinue the study from a medical perspective.

3.3 Number of cases and grouping methods

1226 Adults (18-100 years old) patients with acute limb fractures and received conservative treatment will be randomly divided into group A (intervention group) and group B (control group), 613 of each group. The intervention group will receive oral pills of oxycodone (5 mg)/acetaminophen (325 mg), and the control group will receive pills of acetaminophen (650 mg) only. All participants will be instructed to take one pill of study medication on an as-needed basis, but no more frequently than once every 4 hours. We will routinely provide pain management education and dosage guidance education.

3.3 Drugs

Intervention group (Tylox (acetaminophen/oxycodone)): tablets, dose 325mg acetaminophen + 5mg oxycodone, oral administration, the usual dose is taken every 4 hours, depending on the degree of pain.

Control drug (Tylenol (acetaminophen)): tablets, dose of 650 mg acetaminophen, oral administration, the usual dose is 1 tablet every 4 hours, no more than 4 times within 24 hours.

After generating 1226 processing codes with randomized method and randomly assigned to the test group, the drug was prepared (Hongyi Zhu is responsible for randomly generating 1226 processing numbers, assembling drugs, and completing blind codes and preservation). Each study drug package contains 1 bottle, and each bottle contains 20 pieces of oxycodone/acetaminophen or acetaminophen tablets (according to the blinding code) loaded with fully identical capsules (assisted by our center pharmacy). The outer packaging and the shape of the bottle are exactly the same, and a random number sequence will be attached to the outer packet. Documents with the participant number, drug code, and test group (also known as blind codes) were recorded in duplicate and were deposited with the clinical research unit and the research applicant. An emergency letter is set in each of the completed packets, including study group allocation and medication type contained within the packet, and may not be disassembled if not necessary. In the event of an emergency, serious adverse reaction, or if the participant needs to know what kind of treatment he/she is receiving, researchers and the participant can obtain the information from the emergency letter. If the seal to the emergency letter is opened and letter is read, the participant will be excluded from the trial.

After data collection is completed, a two-step method is used to reveal group membership. The first step lists only the treatment group to which each participant belongs (such as group A or B) but does not indicate which group is the intervention group or the control group. The second step reveals the treatment received by groups A and B, and which group is the control group. Statistical data analyst will also be blinded to participant allocation.

3.4 Research steps and related inspections

During the screening period (before nonoperative treatment with analgesic drugs after limb fractures), their information will be collected by a dedicated research physician. Such as the demographic data, lifestyle habits, and past medical history will be reviewed, and general medical examinations were performed.

Screening will be strictly in accordance with the admission criteria, and after the patient was allowed to enter the study, informed consent will be obtained after the patient had fully informed consent. The participant will be measured with NRS scores, SAS, SDS, and EQ-5d scales. Participants were randomized by random number method, and the study drugs were distributed according to the group arrangement and taken as prescribed.

During the follow-up period (1, 3, 7 days after nonoperative treatment), the participants will complete the NRS score and the change in participant's sleep time ill be recorded. During the follow-up period (14 days after nonoperative treatment), the participants will complete the NRS score, SAS, SDS, the EQ-5d scale and the patient’s satisfaction with the medication (0-10), change in the quality and duration of sleep, number of study medications used, duration that analgesics were taken, and adverse events will be recorded. The first, second and third follow-ups will be followed by telephone, and the last follow-up will be performed on-site.

The drug allocation and follow-up will be carried out by Xiaozhong Zhu, Bingbo Bao, Xingwei Li, Tao Gao et al. Standardized training and assessment will be carried out before the start of the study. Researchers will be eligible for drug allocation, follow-up and data collection.

3.5 Outcomes

Primary outcomes: the between-group difference in decline in NRS pain scores from the baseline to 1, 3, 7, and 14 days after randomization

Secondary outcomes: SAS, SDS, the EQ-5d scale and the patient’s satisfaction with the medication (0-10), change in the quality and duration of sleep, number of study medications used, duration that analgesics were taken, and adverse events

3.6 Standards of suspension

1) Serious adverse events

3.7 Combined medication and treatment

  In this study, you are prohibited from using the following drugs: central nervous system inhibitors, anticholinergics, oral contraceptives, beta blockers, medullary diuretics, lamotrigine, probenecid, and zidovudine and so on.

3.8 Follow-up and medical measures when the trial is over.

  After the study, if there is still analgesia, you can come to our clinic to seek pain management.

1. **Adverse event observation**

4.1 Definition of adverse events

4.1.1 Definition

Adverse events: Adverse medical events that occur after a patient or clinical trial participant receives a drug, but do not necessarily have a causal relationship with treatment.

Severe adverse events: adverse events that require hospitalization, prolonged hospital stay, disability, work ability, life-threatening or death, and congenital malformations during clinical trials.

4.1.2 degree

Mild: Participants can tolerate, do not affect treatment, do not need special treatment, and no effect on the rehabilitation of the participant.

Moderate: The participant is unbearable and requires special treatment, which has a direct impact on the rehabilitation of the participant.

Severe: endangering the life of the participant, causing death or disability, requiring immediate emergency treatment.

4.2 Recording and reporting of adverse events

Participants may experience clinical adverse events during treatment. In the event of adverse events (including severe adverse events), the time, clinical performance, treatment and duration, and outcome of adverse events should be recorded in the case report form; In the case of abnormal laboratory tests, patients should be followed up until the results return to normal, or to the pre-dose level, or determined to be independent of the test drug. In the event of serious adverse events, the serious adverse event report form should be completed and the sponsor, ethics committee, CFDA Safety Supervision Department and health administration department should be reported within 24 hours.

4.3 Risk prevention and treatment

Prevention of risk: Before the participant signs the informed consent form, explain to the participant in detail the possible adverse reactions and manifestations of the drugs used in the test, and educate them how to use the drug, etc.; And if adverse reactions occur, they are actively treated regardless of whether they are related to the drugs used. The costs incurred in the processing of damage associated with this study will be the responsibility of the investigator.

1. **Statistical Analysis**

5.1 sample size estimates

The sample size was calculated by using the following parameters. An overall 2-sided significance level of 0.05, power of 90% will be calculated. In line with previous studies, only if the between-group change difference of NRS scores is reached 1.3 unit or great, it seems as clinically significant in pain,[15] and the standard deviation of the difference in NRS scores between the two groups was 6.4 points from our prior work.

Uα (0.05)=1.960，Uβ（0.1）=1.282， S=6.4， δ=1.3

Substituting into the formula: NA=NB=2×[(1.960+1.282) ×6.4/1.3]2=511

Taking into account factors like patients loss-to-follow-up and participant withdrawal during the trial, it is reasonable to increase the sample size by 20%. The sample size of each arm is estimated to be about 613, and the total sample size required for this clinical trial is 1226

5.2 Statistics and analysis of research data

After the trial, the research data was submitted to a third-party statistical analyst for analysis. The dates will be processed by SPSS16.0 statistical software. The measurement data will be expressed as mean. The t test was used for comparison between groups. P<0.05 was considered statistically significant.

1. **Research related ethics**

6.1 Ethics Committee Review

This protocol and written informed consent and information directly related to the participant must be submitted to the Ethics Committee for approval. This study won’t start until obtained the approval of the Ethics Committee. The investigator must submit an annual research report to the Ethics Committee at least annually (if applicable). At the time of the study's suspension and/or completion, the investigator must notify the ethics committee in writing; the investigator must promptly report to the ethics committee any changes that have occurred in the research work (such as revisions to the program and/or informed consent), and without ethics These changes may not be performed until the committee approves, except to make changes to the obvious and immediate risks to the participant. In the event of such a situation, the Ethics Committee will be notified.

6.2 Informed consent

6.2.1 Procedure for obtaining informed consent

The investigators must provide the participant or his or her legal representative with an informed consent and informed consent from the ethics committee and give the participant or his or her legal representative sufficient time to consider it. Participants may not be enrolled until they have signed the written informed consent form. All updated versions of informed consent and written information will be provided to the participant during the participant's participation. Informed consent should be kept as an important document for clinical trials.

1. **Data privacy and security monitoring program**

The results of the research through this project may be published in medical journals, but we will keep the patient's information confidential, and the patient's personal information will not be disclosed unless the law requires. When necessary, government management departments and hospital ethics committees and their related personnel can access the patient's information as required.

1. **Quality control and quality assurance in clinical research**

Before the start of the trial, develop a standard operating procedure (SOP) for clinical trials, conduct relevant training for all participants, and carefully check the implementation of the SOP at the beginning of the trial, and systematically apply the SOP for its applicability and effectiveness during the trial. Check and modify and supplement the SOPs that are not applicable.

Each work in the clinical trial process is based on the relevant regulations and management regulations, job responsibilities, technical specifications of the work, and the requirements of the test program to develop standard operating procedures for the work.

Study participants and variables are clearly defined, and study participants are randomly selected and assigned. The double-blind test was used to ensure the balance between the test group and the control group; the testers were called to conduct unified technical training and technical assessment.
